# Fracture of the Fabella: A Rare Injury in Knee Trauma

**DOI:** 10.1155/2012/390150

**Published:** 2012-11-20

**Authors:** Andre Rodrigues Façanha Barreto, Francisco Abaete Chagas-Neto, Michel Daoud Crema, Mario Muller Lorenzato, Mariana Tiemi Teixeira Kobayashi, Carlos Ribeiro Monteiro, Marcello Henrique Nogueira-Barbosa

**Affiliations:** ^1^Division of Radiology, Department of Internal Medicine, Ribeirão Preto School of Medicine, University of São Paulo, 14030-000 Ribeirao Preto, SP, Brazil; ^2^School of Medicine, Boston University, Boston, MA 02215, USA

## Abstract

We present a case of a 21-year-old woman sustaining a traumatic [fabellar] fracture following a motor vehicle accident. The fabellar fracture was confirmed on plain films, which prompted further evaluation of the knee with ultrasound and magnetic resonance imaging to evaluate other possible associated injuries. Fracture of the fabella is a rare condition. Clinically, patients present with posterolateral knee pain, edema, and limited knee extension. Occasionally these symptoms may be very subtle, delaying the correct diagnosis and patient management.

## 1. Introduction

 The fabella is a fibrocartilaginous or ossified sesamoid bone of the knee, classically located within the tendon of the lateral head of the gastrocnemius muscle, but may rarely be found within the medial head of that muscle [1, 2]. 

 Its ossification pattern is endochondral, either partial or total, occurring around 12–15 years of age, later than patella's ossification. The estimated overall prevalence varies from 10% to 30%, reaching 66% in some reported series [2, 3]. In nonpathologic conditions, bony fabellae may measure up to 15 mm [2, 3].

 The fabella may be involved in a variety of pathological entities. Fabellar fracture, either secondary to direct trauma or to chronic stress, is a rare entity being frequently underdiagnosed [4–8].

## 2. Case Presentation

**Figure 1:**
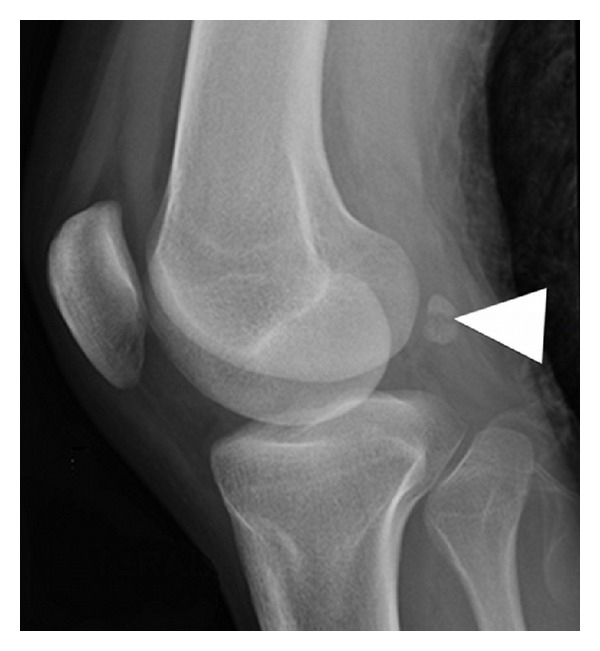
Lateral plain film of the left knee revealed a transverse radiolucent line with irregular borders across an ossified fabella (arrowheads) consistent with a complete fracture of the fabella.

 A previously healthy 21-year-old woman riding on the back seat of a vehicle with a fasten seat belt was involved in a car accident, leading to a direct left knee trauma. After initial assessment at the emergency department, she complained of diffuse left knee pain, which was aggravated by passive extension and palpation, mostly around the posterolateral corner. Anteroposterior and lateral plain films of the left knee were then taken for initial evaluation (Figure 1) and revealed a transverse radiolucent line with corrugated borders across an ossified fabella—regarded as a complete fracture—and also a small avulsion cortical fracture at the medial aspect of the medial femoral epicondyle. An ultrasound of the knee was performed to assess the superficial tendons and ligaments around the knee, especially at the posterolateral corner, as well as to confirm the presence of joint effusion. Posterolaterally, the fabella was depicted with a central cortical defect related to the fabellar fracture seen on radiographs. To further evaluate and rule out injury of the internal structures of the knee, a routine magnetic resonance imaging (MRI) of the left knee was performed.

**Figure 2:**
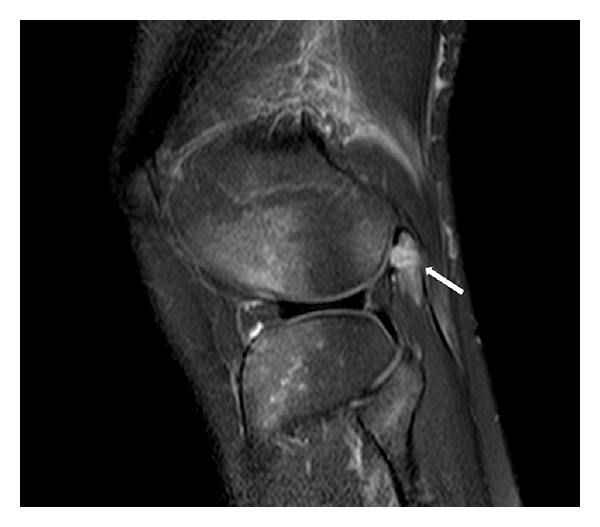
A sagittal T2-weighted fat-suppressed MRI demonstrated diffuse bone marrow edema pattern of the fabella (white arrow) associated with fracture seen on radiographs.

**Figure 3:**
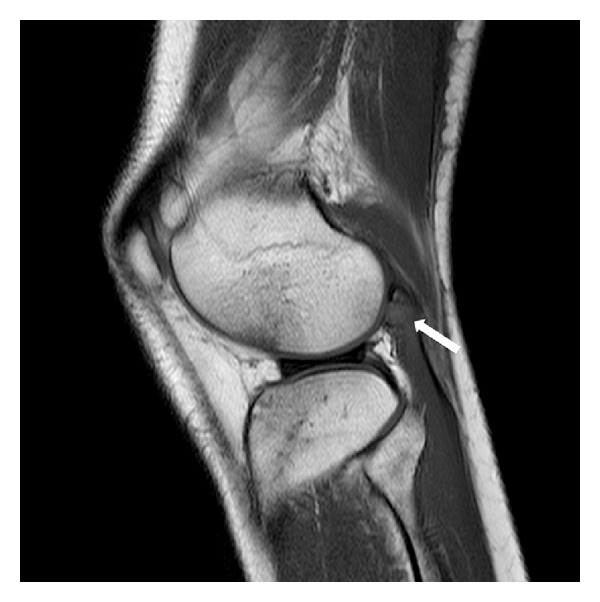
T1-weighted fast spin-echo MRI showed bone contusions in the anterior femoral and tibial regions at the lateral tibiofemoral compartment, related to hyperextension and valgus mechanisms of injury.

**Figure 4:**
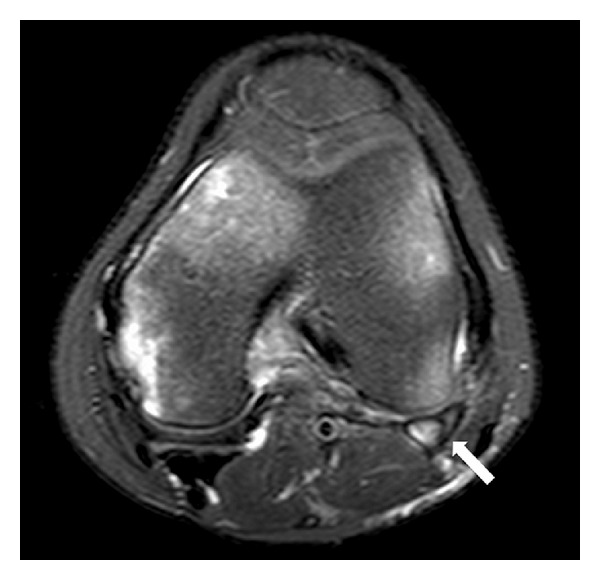
Axial T2-weighted fat-suppressed MRI depicted a low signal line within the fabella consistent with fracture (white arrow).

 MRI was performed in a 1.5 Tesla scanner using sagittal T1-weighted fast spin-echo and triplanar intermediate-weighted fast spin-echo fat suppressed sequences, which confirmed the transverse fracture of the fabella associated with edema-like changes within the bone marrow and within the proximal fibers of the lateral head of gastrocnemius (Figures 2, 3, and 4). Multiple foci of bone edema-like changes consistent with bone contusions at the femoral condyles, tibial plateau, and the fibular head were also detected. The avulsion fracture of the medial aspect of the medial femoral epicondyle, located close to the insertion of medial collateral ligament, was also confirmed. No other abnormalities were detected.

 A conservative management strategy and symptomatic treatment for pain were adopted in the acute setting for this case, as no definitive surgical indication was present. At three months followup, the patient has recovered well and has no current knee complaints.

## 3. Discussion 

 The fabella usually forms a typical synovial joint with the lateral femoral condyle [3]. This sesamoid plays a major biomechanical role in the knee as it consists of a point of confluent forces, and that is postulated as the reason why there is a greater rate of ossification of laterally positioned fabellae [1, 3].

 The fabella may contribute to the stabilization of the posterolateral knee corner and its formation is directly related to the presence and degree of development of the bony fabella [3, 9]. 

 The fabella can be involved in a number of pathological conditions at different age groups [10, 11]. Among those conditions, the most reported are traumatic and stress fractures of the fabella, total knee arthroplasty-related fabellar impingement or snapping [2, 6], chondromalacia fabellae [7, 10], tendinitis of the lateral head of the gastrocnemius muscle, primary osteoarthritis, compression of the thickened gastrocnemius tendon against the femoral condyle, and peroneal nerve irritation/compression [1, 8].

 Fabellar fractures are rare and may be underdiagnosed. It may occur at all ages and be related to different mechanisms, ranging from direct trauma, such as in the above-presented case, to more chronic stress forces, like impingement after total knee replacement [1, 4]. Considering the low prevalence rates and the almost always subtle clinical findings related to fabellar fractures, it is quite a diagnostic challenge to properly evaluate these lesions. 

 When suspected, it is possible to obtain an early diagnosis and an adequate management of cases to prevent morbidity, which is mainly related to knee pain and functional impairment. Fabellar fracture by itself may be conservatively managed in the acute setting, as in our case. But one must keep in mind that whenever a fabellar fracture is suspected, there should be a concern to evaluate for potentially associated lesions that may require surgical treatment. 

 Further, the healing process may produce morphologic changes in the fabella that could lead to chronic conditions, such as an enlarged osteoarthritic fabella, which has been related to fabellar dislocation and peroneal nerve injuries, all potential sources of chronic knee pain [5, 8, 11].

 We presented an unusual case of a fabellar fracture following direct knee trauma. Radiologists must be aware of such condition and consider it in the differential diagnosis of pain in the posterolateral corner of the knee following trauma.
